# Structured continuous positive airway pressure weaning standardizes discontinuation and reduces instability events

**DOI:** 10.3389/fped.2026.1776103

**Published:** 2026-03-11

**Authors:** Philipp Deindl, Juliane Nowotni, Hanna Maruhn, Eik Vettorazzi, Dominique Singer, Mandy Lange

**Affiliations:** 1 Department of Neonatology and Pediatric Intensive Care Medicine, University Children’s Hospital, University Medical Center Hamburg-Eppendorf, Hamburg, Germany; 2 Department of Pediatrics and Adolescent Medicine, Hospital of Lüneburg, Lüneburg, Germany; 3 Department of Medical Biometry and Epidemiology, University Medical Center Hamburg-Eppendorf, Hamburg, Germany

**Keywords:** clinical protocol, CPAP, neonatal intensive care, non-invasive respiratory support, preterm infants, weaning

## Abstract

**Background/objectives:**

Continuous positive airway pressure (CPAP) is a cornerstone of neonatal respiratory support, yet weaning practices remain highly variable and largely experience-based. This study evaluated whether implementing a structured, stage-based CPAP-weaning protocol could standardize clinical decision-making and reduce exposure to poorly tolerated CPAP-off periods, using safety-relevant instability markers.

**Methods:**

In this single-centre interventional study with a historical control group, all neonates reaching ≥30 + 0 weeks corrected gestational age who received CPAP between March 2022 and February 2024 were included. The intervention introduced a seven-stage, pause-based CPAP-weaning protocol with predefined escalation criteria, bedside “monitor cards,” and electronic documentation. Primary endpoints were daily episodes of oxygen desaturation (SpO₂ < 85%) and bradycardia (heart rate < 100 bpm). Secondary endpoints included stimulation frequency, duration of respiratory support, and adverse events.

**Results:**

A total of 344 neonates were analyzed (169 pre-implementation and 175 post-implementation), representing 2,950 CPAP-weaning days. Following implementation, the median number of bradycardia episodes per day decreased [3 [IQR 1–5] vs. 2 [IQR 1–5]; *p* = 0.0004], as did oxygen desaturations [34 [IQR 11–76] vs. 30 [IQR 9–67]; *p* = 0.021]. Stimulation frequency, CPAP duration, and NICU length of stay remained unchanged. Air leak syndromes occurred less frequently after implementation (13% vs. 4%; *p* = 0.011). Protocol adherence was consistently high throughout the study period.

**Conclusions:**

Implementation of a structured CPAP-weaning protocol was associated with fewer daily instability events without prolonging respiratory support. These findings most likely reflect improved safety and standardization of CPAP discontinuation rather than enhanced intrinsic respiratory stability, supporting the feasibility of protocolized weaning in routine NICU care.

## Introduction

Continuous positive airway pressure (CPAP) is a cornerstone of neonatal respiratory care and has substantially improved outcomes in preterm and term infants with respiratory distress. Despite its widespread use, the process of discontinuing CPAP remains highly variable across neonatal intensive care units (NICUs) and is largely guided by local experience rather than standardized criteria. This variability may influence both treatment duration and patient safety.

Previous studies have shown that the lack of structured weaning approaches contributes to inconsistent clinical decision-making and may affect respiratory stability during the transition to spontaneous breathing (1–3). Although several weaning strategies, such as gradual pressure reduction or intermittent CPAP-off periods, have been proposed, evidence regarding their comparative effectiveness remains inconclusive (4–9). In daily clinical practice, decisions are often based on subjective assessments of stability, varying staff experience, and institutional routines, all of which can lead to substantial heterogeneity in care delivery (8, 10, 11).

To address this gap, a structured, pause-based CPAP-weaning protocol was developed and implemented in a level IV NICU in Germany. The protocol consisted of seven predefined stages with gradually increasing CPAP-off periods, explicit escalation and discontinuation rules, and a bedside monitoring tool (“monitor card”) integrated into the electronic medical record. Staff training was supported by eLearning modules to ensure consistent application across shifts. The protocol aimed to standardize bedside decision-making during CPAP discontinuation by defining explicit stages, escalation thresholds, and stop rules, rather than to modify underlying respiratory physiology.

This study evaluated the association between protocol implementation and safety-relevant instability markers during CPAP weaning using a historical control design. We hypothesized that protocolization would reduce variability in weaning decisions and shorten exposure to poorly tolerated CPAP-off periods, and result in fewer documented instability events per weaning day under standardized decision rules, without prolonging overall respiratory support.

## Materials and methods

### Study design and setting

This single-centre, retrospective–prospective interventional study with a historical control group was conducted at the Department of Neonatology and Pediatric Intensive Care Medicine, University Medical Center Hamburg-Eppendorf (UKE), Germany, a level IV perinatal centre. The study evaluated the impact of implementing a structured, stepwise CPAP-weaning protocol on clinical stability in neonates.

The intervention was introduced on March 1, 2023. Data were collected for one year before (March 1, 2022 to February 28, 2023) and one year after protocol implementation (March 1, 2023 to February 29, 2024). The study was approved by the Ethics Committee of the Hamburg Chamber of Physicians (reference WF-157/20).

### Participants

All neonates treated with CPAP during the study periods were screened for eligibility. Infants across the full spectrum of gestational ages at birth, including term neonates, were eligible for inclusion, provided they received CPAP and reached a corrected gestational age of at least 30 + 0 weeks during their NICU stay, which defined the threshold for application of the structured CPAP-weaning protocol. Exclusion criteria included major congenital anomalies, hydrops fetalis, syndromic diagnoses, birth asphyxia, and cardiopulmonary resuscitation at birth, as these factors could independently affect respiratory stability and weaning trajectories.

### Intervention: structured CPAP-weaning protocol

The structured weaning protocol comprised seven predefined stages (Stage 0–6) characterized by CPAP-off periods of increasing duration (see Supplementary Figure 1). The initial stage was determined by gestational age and clinical condition, for example, no pauses for infants ≤ 29 + 0 weeks, within 24 h after less-invasive surfactant administration (LISA) or extubation, or when PEEP exceeded 5 cm H₂O. Infants born at 29 + 0–30 + 6 weeks started with Stage 1, and those ≥ 31 + 0 weeks with Stage 3. Progression through the stages ranged from short 15–20 min pauses during nursing care (Stage 1) to complete discontinuation (Stage 6). Each weaning stage did not correspond to a predefined fixed duration but was defined by alternating CPAP-off intervals. Progression to the next stage was assessed based on predefined clinical stability criteria and could occur multiple times per day. Accordingly, infants were able to complete several weaning stages within 24 h, and complete discontinuation of CPAP within one or two days was possible in clinically stable infants, particularly when entering the protocol at intermediate stages.

Infants who had previously received invasive mechanical ventilation or non-invasive ventilation modes such as biphasic positive airway pressure (BIPAP/NIPPV) were transitioned to CPAP according to standard unit practice before entering the structured CPAP-weaning protocol. The CPAP-weaning strategy itself was identical for all infants, irrespective of the type or duration of preceding respiratory support.

At least once daily and during each CPAP-off period, clinical stability was assessed using predefined criteria for oxygen requirement, respiratory rate, work of breathing, and the number and severity of desaturation or bradycardia episodes (see Supplementary Table 1). These were categorized as stable, tolerable, or unstable. Advancement to the next stage required documentation of stability or tolerability, while instability mandated continuation at the current stage.

A bedside “monitor card” (Supplementary Figure 2) was used to document all respiratory events during CPAP-off phases. Mild events were defined as transient desaturations or bradycardias with spontaneous recovery; severe events required stimulation such as tactile support, oxygen flush, or manual inflation (clinician-triggered ventilation breath delivered via the ventilator using predefined pressure settings, without disconnecting the infant from the CPAP or ventilation interface); threatening events required major intervention such as bag-mask ventilation (manual ventilation performed after disconnecting the CPAP interface, using a self-inflating bag and face mask). Pauses were discontinued when escalation criteria were reached (three or more severe or ten or more mild events per hour, or any threatening event). All documentation was integrated into the electronic patient record to ensure consistent bedside application.

### Data collection

Demographic and perinatal characteristics were extracted from the electronic medical record, including birth weight, gestational age, sex, mode of delivery, and maternal chorioamnionitis. Treatment-related data included duration of respiratory support, surfactant administration, and use of LISA. Staff experience (years in neonatal care, completion of pediatric intensive care training) was also documented.

The primary endpoints were the number of oxygen desaturation episodes (SpO₂ < 85%) and bradycardias (heart rate < 100 bpm) and were assessed per 24-hour CPAP-weaning day, independent of whether infants were on or off CPAP at the time of event occurrence. These endpoints were defined *a priori* as primary outcomes because they represent continuous, safety-relevant markers of respiratory instability that are closely linked to CPAP-off periods and are independent of clinician decision-making. Secondary endpoints included stimulation frequency, duration of CPAP and invasive ventilation, incidence of air leak syndromes and other complications, daily weight gain during CPAP therapy, and NICU length of stay. Secondary outcomes were selected to capture broader treatment trajectories and resource-related effects, but were not expected to be directly modified by protocolization of CPAP weaning. Only infants with at least one documented CPAP-weaning day were included in the per-protocol analysis.

Continuous bedside monitoring was performed using the Philips IntelliVue system (Philips Healthcare, Amsterdam, The Netherlands). Vital parameters were automatically imported into the Integrated Care Manager (ICM, Dräger, Lübeck), where desaturation and bradycardia events were validated by nursing staff and incorporated into the patient chart. This acquisition and validation process was identical in the pre- and post-intervention phases, ensuring consistent measurement of primary endpoints across study periods. Post-intervention, documentation of CPAP weaning stages was supported by a structured electronic tool that standardized recording of weaning progression but did not affect the acquisition or validation of physiological events.

### Statistical analysis

All analyses were conducted using R version 4.3.3 (R Core Team, 2023) within a reproducible R Markdown workflow. Continuous outcome variables were summarized using medians and interquartile ranges (IQR), as event counts per CPAP-weaning day showed non-normal distributions. Group comparisons between pre- and post-implementation phases were performed using the Wilcoxon rank-sum test. Multivariable linear regression models were constructed to identify predictors of clinical stability, defined as the frequency of desaturations and bradycardias. Predictor variables included study phase (pre- vs. post-implementation), use of LISA, gestational age, and sex. Staff experience was excluded from the final models due to lack of association with outcomes. A two-sided *p* value below 0.05 was considered statistically significant. All data were pseudonymised prior to analysis. The full dataset and analytic code are available upon reasonable request as outlined in the data availability statement.

## Results

### Study population

During the study period, 555 neonates were treated in the NICU in the year before and 530 in the year after protocol implementation. Of these, 113 infants (20.4%) in the pre-intervention cohort and 113 infants (21.3%) in the post-intervention cohort required invasive mechanical ventilation. Non-invasive ventilation, including nasal intermittent positive pressure ventilation (NIPPV), CPAP, or high-flow oxygen therapy, was applied in 214 infants (38.6%) before and 197 infants (37.2%) after implementation. Infants who had previously required invasive ventilation were subsequently supported with CPAP, whereas others received CPAP as their primary mode of respiratory support. In neonatal care, the term “weaning” refers to the gradual transition from CPAP to autonomous breathing, independent of prior invasive ventilation.

**Figure 1:**
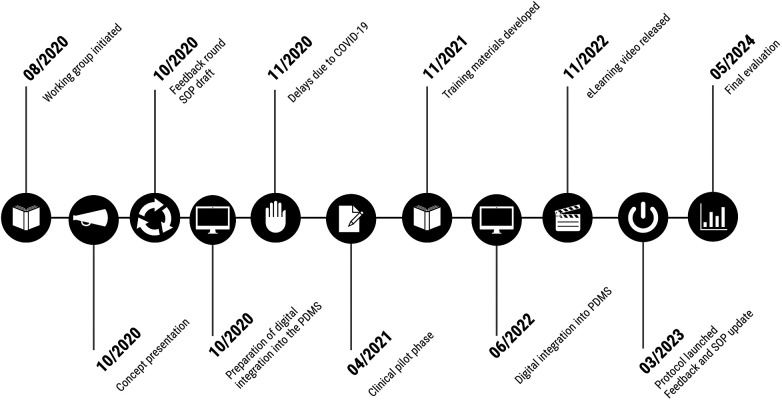
Timeline of key milestones in the development and implementation of the structured CPAP-weaning protocol. The figure illustrates major steps from the initial concept and training phase to the full clinical integration of the protocol into the patient data management system (PDMS). CPAP, continuous positive airway pressure; SOP, standard operating procedure; PDMS, patient data management system.

A total of 344 patients met inclusion criteria and were analysed in detail, comprising 169 infants before and 175 after protocol implementation. Table 1 presents the baseline demographic and clinical characteristics of the study population. A timeline of protocol development and implementation is shown in Figure 1.

**Table 1:** Baseline demographic and clinical characteristics of the study population.

Characteristic	Before	After	*P*-Value
	*n* = 169	*n* = 175	
Birth weight (kg)	2.072 (1.74–2.8)	2.02 (1.58–2.84)	0.719
Small for gestational age	8 (5)	18 (10)	0.128
Head circumference at birth (cm)	31 (28–33.5)	30.5 (28–33)	0.921
Body length at birth (cm)	43 (39–49)	43 (39–48)	0.663
Gestational age (days)	232.5 (212.5–258)	230 (212–256)	0.987
5-minute Apgar score	8 (7–9)	8 (7–9)	0.261
Spontaneous vaginal delivery	124 (73)	122 (70)	0.527
Female sex	65 (38)	81 (46)	0.174
Maternal chorioamnionitis	25 (15)	42 (24)	0.098

Continuous variables are presented as median (Q1–Q3), and categorical variables as absolute numbers with percentages in parentheses. Group comparisons of continuous variables were performed using the two-tailed Wilcoxon rank-sum test with continuity correction. Categorical variables were analyzed using the two-tailed chi-square test. CPAP, continuous positive airway pressure.

### Protocol uptake

**Figure 2:**
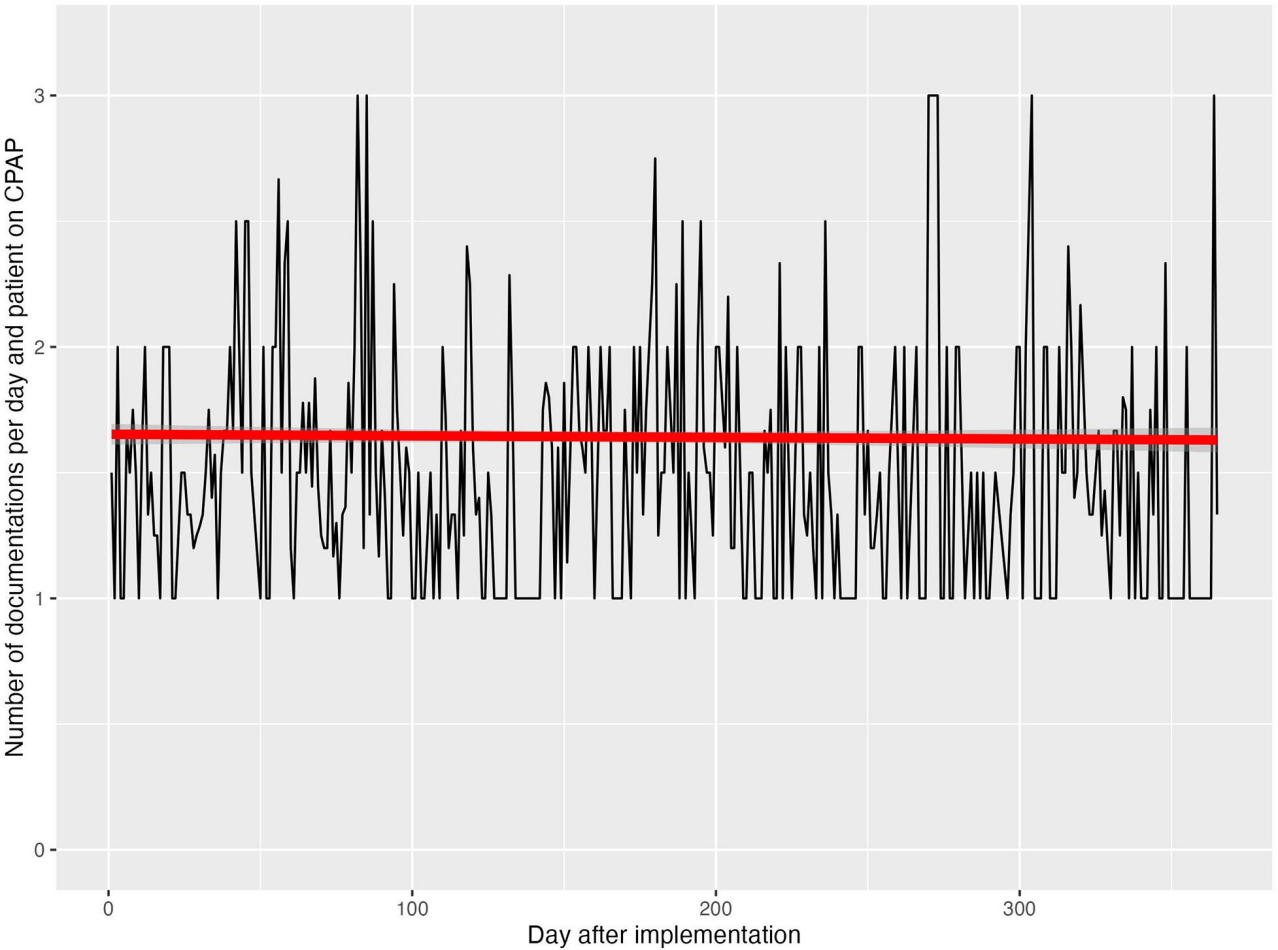
Uptake of the structured CPAP-weaning protocol after implementation. Average number of documented weaning stage entries per patient-day during the first 12 months after implementation. At least one daily documentation was defined as the minimal quality target, corresponding to the daily multidisciplinary ward round. Additional entries occurred when clinically relevant changes arose during the day. Documentation frequency reflects protocol uptake and integration into routine clinical workflow.

Protocol uptake was assessed by the frequency of documented weaning stage entries per patient-day. On average, 1.6 ± 0.5 weaning stages were documented per patient per day, indicating regular use of the protocol in daily clinical routine. While documentation of weaning stages was consistently performed, detailed assessment of adherence to all escalation and stop rules was not feasible retrospectively for every weaning episode. Figure 2 illustrates the integration of the structured weaning protocol into routine documentation.

### Instability events during CPAP weaning

**Figure 3:**
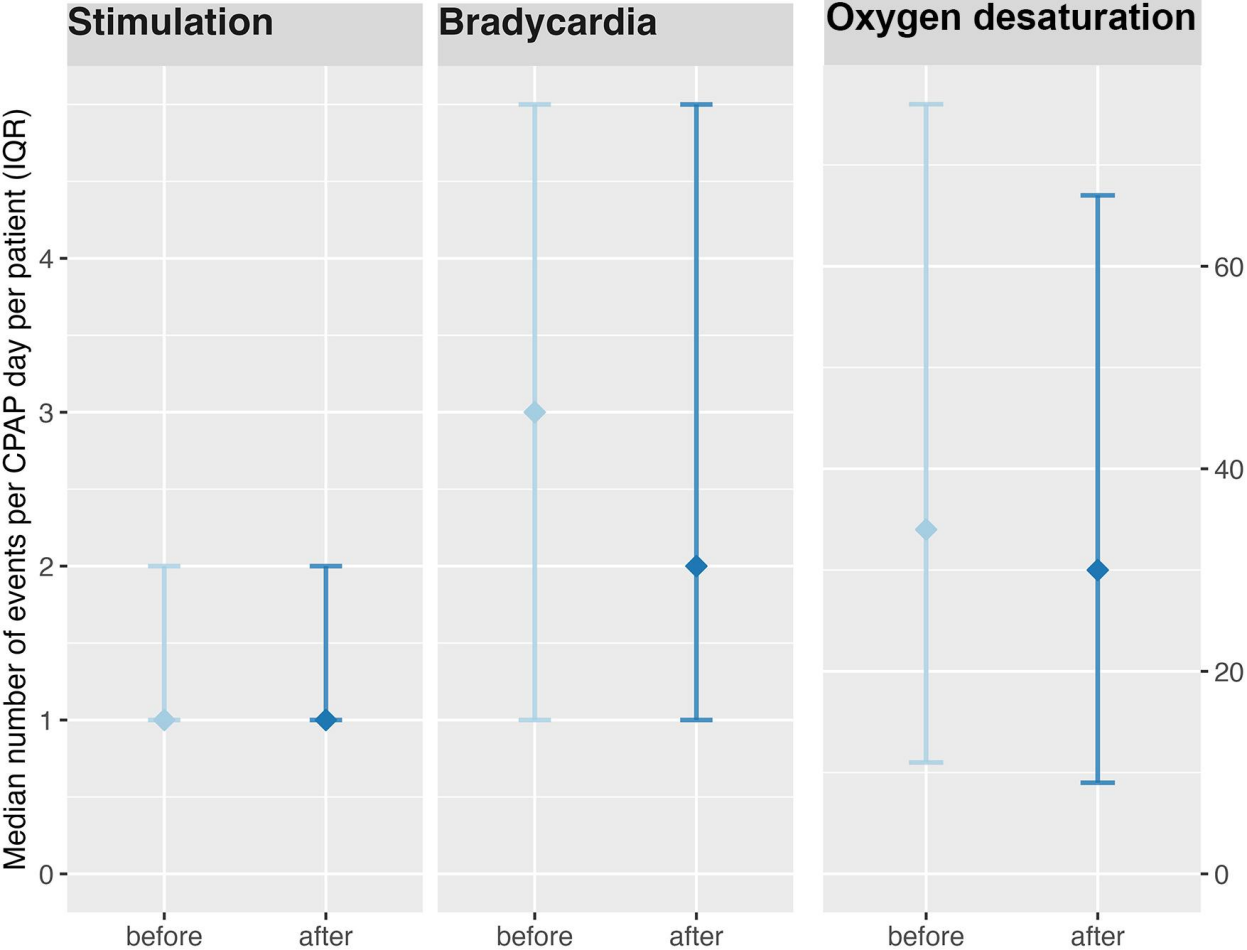
Clinical stability before and after implementation of the structured CPAP-weaning protocol. Median values with interquartile ranges (Q1–Q3) are shown for stimulation events, bradycardias, and oxygen desaturation episodes (SpO₂ < 85%) per CPAP-weaning day. Data include 1,468 weaning days before and 1,482 weaning days after implementation. CPAP, continuous positive airway pressure; SpO₂, peripheral capillary oxygen saturation.

A total of 2,950 CPAP-weaning days were analysed, including 1,468 days before and 1,482 days after implementation. The median number of bradycardia episodes per CPAP-weaning day was lower after protocol implementation compared with the pre-implementation phase [median 2 [IQR 1–5] vs. 3 [IQR 1–5]]. This difference was statistically significant in non-parametric analysis (*p* = 0.0004). Similarly, the median number of oxygen desaturation episodes (SpO₂ < 85%) per CPAP-weaning day decreased following implementation of the structured weaning protocol [median 30 [IQR 9–67] vs. 34 [IQR 11–76]]. The difference reached statistical significance (*p* = 0.021) (Figure 3).

### Secondary endpoints: respiratory support, hemodynamic support, and growth

The median number of stimulation events per CPAP-weaning day remained unchanged between study phases [median 1 (IQR 1–2) in both groups; *p* = 0.10]. The duration of CPAP support did not differ between groups [median 2.0 [IQR 1.0–11.0] vs. 3.0 [1.0–13.0] days; *p* = 0.569]. Similarly, no significant differences were observed in the duration of invasive ventilation [median 3.0 [2.0–9.5] vs. 3.0 [2.0–5.3] days; *p* = 0.611], non-invasive intermittent positive pressure ventilation [median 5.0 [1.5–11.5] vs. 3.0 [1.0–8.0] days; *p* = 0.550], or high-flow nasal cannula therapy [median 3.0 [2.0–10.5] vs. 4.0 [3.0–10.3] days; *p* = 0.493].

Hemodynamic support, as reflected by the duration of catecholamine therapy, was comparable between groups [median 2.0 (2.0–4.0) days in both cohorts; *p* = 0.817]. Growth-related outcomes showed no significant differences, including weight at the end of CPAP weaning [median 2.072 [1.74–2.80] vs. 2.020 [1.58–2.84] kg; *p* = 0.298] and daily weight gain among infants receiving CPAP therapy for at least ten days [median 0.0 [−5.9–19.2] vs. 0.31 [−11.7–19.7] g/day; *p* = 0.548].

The duration of CPAP weaning itself was similar between study phases [median 2.0 [1–13] vs. 3.0 [1–14.7] days; *p* = 0.695]. Likewise, the length of NICU stay [median 4.0 [1–21.5] vs. 6.0 [2–23.8] days; *p* = 0.124] and total hospital stay [median 25.4 [7.9–48.1] vs. 26.3 [8.9–43.1] days; *p* = 0.978] did not differ significantly (Table 2).

**Table 2:** Clinical outcomes before and after implementation of the structured CPAP weaning protocol.

Variable	Before	After	*P*-Value
	*n* = 169	*n* = 175	
Delivery room management			
Intubation	50 (30)	49 (28)	0.591
Less-invasive surfactant administration	14 (8)	52 (30)	**<0**.**001**
Surfactant administration	51 (30)	81 (46)	**0**.**005**
Respiratory and hemodynamic support and growth in the NICU			
CPAP support (days)	2.0 (1.0–11.0)	3.0 (1.0–13.0)	0.569
High-flow nasal cannula (days)	3.0 (2.0–10.5)	4.0 (3.0–10.3)	0.493
Invasive ventilation (days)	3.0 (2.0–9.5)	3.0 (2.0–5.3)	0.611
Non-invasive intermittent positive pressure ventilation (days)	5.0 (1.5–11.5)	3.0 (1.0–8.0)	0.550
Duration of catecholamine therapy (days)	2.0 (2.0–4.0)	2.0 (2.0–4.0)	0.817
Weight at end of weaning (kg)	2.072 (1.74–2.80)	2.020 (1.58–2.84)	0.298
Daily weight gain during CPAP therapy ≥10 days (g/day)	0.0 (−5.9–19.2)	0.31(−11.7–19.7)	0.548
Duration of CPAP weaning (days)	2.0 (1–13)	3.0 (1–14.7)	0.695
Length of NICU stay (days)	4.0 (1–21.5)	6.0 (2–23.8)	0.124
Length of hospital stay (days)	25.4 (7.9–48.1)	26.3 (8.9–43.1)	0.978
Adverse clinical outcomes			
Pneumothorax/pneumomediastinum	22 (13)	7 (4)	**0**.**011**
Bronchopulmonary dysplasia (BPD)	11 (7)	12 (7)	0.991
Focal intestinal perforation	3 (2)	1 (1)	0.581
Necrotizing enterocolitis requiring surgery	2 (1)	2 (1)	0.999
Intraventricular hemorrhage (grade III or higher)	7 (4)	6 (3)	0.949
Persistent ductus arteriosus requiring surgery	6 (4)	3 (2)	0.566
Persistent ductus arteriosus treated with medication	17 (10)	11 (6)	0.440
Persistent pulmonary hypertension of the newborn (PPHN)	12 (7)	8 (5)	0.605
Periventricular leukomalacia (PVL)	2 (1)	1 (1)	0.830
Retinopathy of prematurity (grade III/IV)	4 (2)	2 (1)	0.649
Death	1 (1)	0 (0)	0.595

Bold values show statistically significant *p*-values (<0.05).

Values are presented as median (Q1–Q3) for continuous variables or as absolute numbers with percentages in parentheses for categorical variables. Group comparisons of continuous variables were performed using the two-tailed Wilcoxon rank-sum test with continuity correction; categorical variables were analysed using the two-tailed chi-square test. CPAP: continuous positive airway pressure; HFNC: high-flow nasal cannula; LISA: less-invasive surfactant administration; BPD: bronchopulmonary dysplasia; NEC: necrotising enterocolitis; IVH: intraventricular haemorrhage; PDA: persistent ductus arteriosus; PPHN: persistent pulmonary hypertension of the newborn; PVL: periventricular leukomalacia; ROP: retinopathy of prematurity.

### Adverse events and complications

The incidence of air leak syndromes decreased significantly after protocol implementation (13% vs. 4%, *p* = 0.011). Other relevant complications, including intraventricular hemorrhage, bronchopulmonary dysplasia, necrotizing enterocolitis, and retinopathy of prematurity, occurred at similar rates in both groups (Table 2). One neonatal death occurred in the control group, whereas no deaths were recorded after implementation. In multivariable regression analyses, both study phase and LISA therapy were associated with differences in clinical stability, while gestational age, sex, and staff experience showed no significant effects.

## Discussion

In this single-centre before–after study, implementation of a structured, stage-based CPAP-weaning protocol was associated with fewer bradycardia and desaturation events per CPAP-weaning day, while overall respiratory support duration and length of stay remained unchanged. Using non-parametric analyses, we observed a shift toward lower daily counts of bradycardia and desaturation events after implementation of the structured CPAP-weaning protocol. These findings indicate fewer instability events per weaning day under standardized decision rules, while escalation-relevant outcomes such as stimulation frequency remained unchanged. The findings add to a growing body of evidence that protocolized respiratory care can improve stability and reduce variability in clinical decision-making.

### Relation to existing evidence on protocolized weaning

Protocolized weaning has a long tradition in adult intensive care. Randomized trials and meta-analyses have shown that structured approaches shorten invasive ventilation and standardize care in adults, although effects vary by setting and implementation quality (1, 3). In neonatology, the evidence base is more heterogeneous and often focuses on specific weaning tactics rather than on system-level protocols.

Trials comparing time-cycling or pause-based strategies with pressure step-down did not demonstrate consistent superiority of one specific tactic across outcomes, but they support the feasibility of structured approaches in preterm infants (4, 6, 11, 12). We chose the pause-based weaning strategy pragmatically, reflecting prevailing local practice and prioritizing feasibility and staff acceptance. The protocol was not intended to establish physiological superiority over pressure-reduction approaches. Instead, it standardized an existing strategy by introducing shared criteria for progression and termination, which may be more relevant for safety and consistency than the specific weaning tactic itself.

Recent syntheses emphasize that non-invasive respiratory strategies are effective but variably applied, and that the comparative effectiveness of weaning methods remains context dependent (5, 7, 9). Variability in NICU CPAP practices, including initiation, weaning, and discontinuation criteria, is well documented and has been identified as a barrier to consistent quality of care (8, 9, 13). The consistent uptake of the protocol, supported by bedside monitor cards and electronic documentation, addresses this gap and suggests that implementation enablers may be as important as the choice of weaning strategy itself.

Protocol uptake was assessed using documentation frequency as a pragmatic process indicator rather than a direct measure of protocol fidelity. Within the structured CPAP-weaning framework, the minimal quality target was defined as at least one documented weaning decision per day, corresponding to the daily multidisciplinary ward round at which respiratory support and weaning status are routinely reviewed. Additional documentation entries were expected only if clinically relevant changes occurred during the day. Given a three-shift nursing system in the NICU, this results in a theoretical maximum of three documentation entries per patient-day. Against this background, an average of 1.6 documented weaning stages per day reflects regular integration of the protocol into daily workflow rather than incomplete adherence (Figure 2). Nevertheless, documentation frequency does not capture compliance with all escalation and stop rules, and further educational or audit-based strategies may be required to improve protocol fidelity.

### Interpretation of reduced instability events

The protocol used intermittent CPAP-off periods at constant PEEP, combined with explicit escalation thresholds and discontinuation rules. Similar pause-based strategies have previously shown comparable treatment durations but more predictable trajectories, particularly when staff follow shared criteria for progression and for aborting pauses (11, 12). Our data replicate this pattern. Following implementation, fewer desaturation and bradycardia events per CPAP-weaning day were observed, while overall respiratory support duration remained unchanged. The observed reduction in daily desaturation and bradycardia events should not be interpreted as improved intrinsic respiratory stability. Rather, it is most plausibly explained by reduced variability in bedside decision-making and earlier recognition of CPAP intolerance during pause-based weaning. By defining explicit escalation thresholds and discontinuation criteria, the protocol likely shortened the duration of poorly tolerated CPAP-off periods, which may have reduced prolonged poorly tolerated pauses, although we could not quantify CPAP-off exposure directly (5, 9).

### Air leak syndromes and interplay with early respiratory care

The lower rate of air leak syndromes after implementation likely reflects two interacting factors. First, structured escalation and stop rules may have reduced episodes of vigorous respiratory effort against insufficient distending pressure during prolonged or poorly tolerated pauses. Second, the post-implementation cohort had more frequent use of surfactant, often via less-invasive surfactant administration, which is associated with improved alveolar stability and reduced barotrauma in preterm infants (11, 14). Network and conventional meta-analyses suggest that strategy bundles integrating appropriate non-invasive support with timely surfactant delivery can improve pulmonary outcomes (7). The higher rate of surfactant administration via less-invasive surfactant administration (LISA) in the post-implementation cohort represents an important confounder. LISA is known to improve pulmonary mechanics and reduce respiratory morbidity and likely contributed to the observed reduction in instability events and air leak syndromes. Multivariable analyses demonstrated associations for both study phase and LISA use, indicating that the effects of protocolization cannot be disentangled from concurrent changes in early respiratory management.

### Contextualization and external validity

Multicentre observational data have highlighted that successful CPAP weaning is influenced by infant maturity and clinical stability, supporting the use of predefined, criterion-based frameworks rather than *ad hoc* decisions (15). Previous studies in neonatology further suggest that structured weaning approaches may reduce practice variability and support more consistent clinical decision-making, even if effects on major clinical outcomes are heterogeneous (16). Our findings are consistent with this body of work and suggest that applying explicit weaning criteria within routine care may contribute to more standardized bedside practice. While supportive tools such as bedside documentation aids and electronic recording were used, the present study was not designed to formally evaluate implementation processes or fidelity. Accordingly, the results should be interpreted primarily in terms of clinical associations and feasibility rather than as evidence of optimized implementation or scalability across settings.

### Clinical implications

For NICUs seeking to standardize CPAP discontinuation, this study supports a low-complexity, pause-based protocol with predefined escalation thresholds, routine daily stability checks, and rigorous documentation. The primary expected benefit is improved safety during CPAP discontinuation through standardized decision-making, not necessarily shorter therapy. In settings with limited resources or high staff turnover, the simplicity of a CPAP-only algorithm may be preferable to equipment-dependent pathways, provided that early surfactant strategies are optimized when indicated (7, 11, 16). The relatively short median CPAP durations observed in this study require contextual interpretation. Importantly, the structured weaning protocol was not based on fixed time intervals per stage but allowed repeated daily advancement contingent on predefined stability criteria. This design enabled clinically stable infants to progress through multiple weaning stages within a short time frame, making complete discontinuation of CPAP within one or two days feasible, particularly for infants entering the protocol at intermediate stages. Consequently, the reported medians primarily reflect a subgroup of rapidly weaned infants with favorable respiratory stability. At the same time, the wide interquartile ranges underscore the substantial interindividual variability inherent to this population and capture infants requiring prolonged respiratory support. These findings highlight that median CPAP duration alone does not represent a uniform weaning trajectory but rather the combined effect of protocol flexibility and heterogeneity in clinical stability.

### Limitations and next steps

This study has several limitations. Its single-centre before–after design precludes causal inference. Although primary endpoints were recorded identically in both study phases, the introduction of structured weaning documentation may have influenced the visibility and consistency of process-related data. Although physiological events were captured consistently across study phases, the electronic documentation did not provide extractable timestamps for CPAP pause start and stop. We therefore could not determine whether individual events occurred during CPAP-off periods or quantify cumulative time off CPAP. This limits mechanistic inference and means that associations should be interpreted at the level of instability events per weaning day rather than pause-specific effects. Additionally, changes in early respiratory management, particularly increased use of LISA, may confound observed associations (7, 11, 14). The absence of a formal power calculation limits inference regarding smaller effect sizes, particularly for secondary outcomes. However, the study was not designed to demonstrate superiority or non-inferiority of a weaning strategy, but to assess whether protocolization of an existing practice was associated with changes in safety-relevant instability markers under real-world conditions.

Future work should test the protocol prospectively across centres, evaluate extremely preterm infants, and compare pause-based CPAP weaning with pressure step-down. Harmonized outcome definitions, including standardized instability composites and patient-centred end points, would facilitate synthesis across studies (5, 7, 9, 16–19).

## Conclusion

In a field marked by heterogeneous CPAP-weaning practices, implementation of a structured, criterion-based protocol was associated with fewer documented desaturation and bradycardia events per CPAP-weaning day without prolonging respiratory support. These findings most likely reflect improved standardization of bedside decision-making and reduced exposure to poorly tolerated CPAP-off periods rather than enhanced intrinsic respiratory stability. Given the before–after design and concurrent changes in early respiratory management, including increased LISA use, causal inference is limited; however, the protocol appears feasible and may support safer, more consistent CPAP discontinuation in routine NICU care.

## Data Availability

The raw data supporting the conclusions of this article will be made available by the authors, without undue reservation.

## Supplementary material

The Supplementary Materialfor this article can be found online at: https://www.frontiersin.org/articles/10.3389/fped.2026.1776103/full#supplementary-material

## References

[1] Krishnan JA Moore D Robeson C Rand CS Fessler HE. A prospective, controlled trial of a protocol-based strategy to discontinue mechanical ventilation. Am J Respir Crit Care Med. (2004) 169(6):673–8. 10.1164/rccm.200306-761OC 14726421

[2] Richardson A Killen AR. How long do patients spend weaning from CPAP in critical care? Intensive Crit Care Nurs. (2006) 22(4):206–13. 10.1016/j.iccn.2005.05.007 16624559

[3] Blackwood B Burns KEA Cardwell CR O’Halloran P. Protocolized versus non-protocolized weaning for reducing the duration of mechanical ventilation in critically ill adult patients. Cochrane Database Syst Rev. (2014) 11:CD006904. 10.1002/14651858.CD006904.pub3 PMC6517015 25375085

[4] Todd DA Wright A Broom M Chauhan M Meskell S Cameron C Methods of weaning preterm babies <30 weeks gestation off CPAP: a multicentre randomised controlled trial. Arch Dis Child Fetal Neonatal Ed. (2012) 97(4):F236–40. 10.1136/adc.2011-300133 22611116

[5] Chowdhury O Wedderburn CJ Duffy D Greenough A. CPAP review. Eur J Pediatr. (2011) 171(9):1441–8. 10.1007/s00431-011-1648-6 22173399

[6] Amatya S Macomber M Bhutada A Rastogi D Rastogi S. Sudden versus gradual pressure wean from nasal CPAP in preterm infants: a randomized controlled trial. J Perinatol. (2017) 37(6):662–7. 10.1038/jp.2017.10 28230835 PMC5446290

[7] Isayama T Iwami H McDonald S Beyene J. Association of noninvasive ventilation strategies with outcomes of preterm infants: a systematic review and network meta-analysis. JAMA Pediatr. (2016) 170(12):1121–30. 10.1001/jama.2016.10708 27532916

[8] Ho JJ Subramaniam P Davis PG. Continuous positive airway pressure (CPAP) for respiratory distress in preterm infants. Cochrane Database Syst Rev. (2020) 10(10):CD002271. 10.1002/14651858.CD002271.pub3 33058208 PMC8094155

[9] Mukerji A Shah PS Shivananda S Yee W Read B Minski J Fusch C; Canadian neonatal network investigators. Survey of noninvasive respiratory support practices in Canadian neonatal intensive care units. Acta Paediatr. (2017) 106(3):387–93. 10.1111/apa.13644 27783410

[10] Mamidi K Bloom J Misra A Rehan VK Lakshminrusimha S. Nasal CPAP practices in neonatal intensive care units in the United States: variability in initiation, weaning, and discontinuation. J Perinatol. (2023) 43(6):703–10.

[11] Kakkilaya V Gautham KS. Should less invasive surfactant administration (LISA) become routine practice in US neonatal units? Pediatr Res. (2023) 93(5):1188–98. 10.1038/s41390-022-02265-8 35986148 PMC9389478

[12] Rastogi S Wong W Gupta A Bhutada A, Deepa Rastogi, Maimonides Neonatal Group. Gradual versus sudden weaning from nasal CPAP in preterm infants: a pilot randomized controlled trial. Respir Care. (2013);58(3):511–6. 10.4187/respcare.01999 22906960

[13] Mukerji A Shah PS Kadam M Borhan S Razak A. Non-invasive respiratory support in preterm infants as primary mode: a network meta-analysis. Cochrane Database Syst Rev. (2025) 7(7):CD014895. 10.1002/14651858.CD014895.pub2 40590276 PMC12210345

[14] Härtel C Kribs A Göpel W Dargaville P Herting E. Less invasive surfactant administration for preterm infants - state of the art. Neonatology. (2024) 121(5):584–95. 10.1159/000540078 39226881 PMC11446307

[15] Bünte LM Walden C Schlecht J Bubl B Popa-Todirenchi MH Tippmann S Early successful weaning from continuous positive airway pressure in infants<32 weeks of gestation: predictors of success. Front Pediatr. (2025) 13:1568891. 10.3389/fped.2025.1568891 40248022 PMC12003399

[16] Mattikalli SN Wisecup K Stephens H Donnelly A Erkinger J Pradhan S Implementation of nasal CPAP weaning guidelines in preterm infants. Respir Care. (2025) 70(2):234–42. 10.4187/respcare.11915 39038832

[17] Balhareth A Khashaba M El-Sayed Y Al-Shehri A Alshehri S. High-flow nasal cannula versus CPAP for weaning preterm infants: a systematic review and meta-analysis. Pediatr Pulmonol. (2024) 59(3):670–9.

[18] Bruet S Butin M Dutheil F. Systematic review of high-flow nasal cannula versus continuous positive airway pressure for primary support in preterm infants. Arch Dis Child Fetal Neonatal Ed. (2022) 107(1):56–9. 10.1136/archdischild-2020-321094 34016651

[19] Katheria A Ines F Hough J Rich W Morales A Sanjay S Changes in lung aeration with high-flow nasal cannula compared to nasal CPAP in preterm infants. J Perinatol. (2025) 45(6):817–22. 10.1038/s41372-025-02267-4 40122991

