# Supplementary material for: Structured continuous positive airway pressure weaning standardizes discontinuation and reduces instability events
Source: Front Pediatr. 2026 Mar 11;14:1776103. doi: 10.3389/fped.2026.1776103 (PMC13013428; doi:10.3389/fped.2026.1776103)
Supplement: Supplementary file 1 [file Table1.pdf]

Supplementary Material Table 1: Stability Assessment During Pause

Parameter	Stable	Tolerable	Unstable
Oxygen requirement	21% / 0 $L\cdot min^{-1}$	22–25% or 0.1–0.5 $L\cdot min^{-1}$	>25% or >0.5 $L\cdot min^{-1}$
Respiratory rate	30–60 min^{-1}	<60 or 60–100 (feeding excluded)	>100 (feeding excluded)
Work of breathing	Normal	Mild retractions, nasal flaring, periodic breathing	≥2 signs, grunting, seesaw breathing
Events during CPAP pause (per h)	<3 severe or <10 mild	3 severe or 10 mild	>3 severe or >10 mild
