# Supplementary material for: Structured continuous positive airway pressure weaning standardizes discontinuation and reduces instability events
Source: Front Pediatr. 2026 Mar 11;14:1776103. doi: 10.3389/fped.2026.1776103 (PMC13013428; doi:10.3389/fped.2026.1776103)
Supplement: Supplementary file 2 [file Image1.pdf]

Supplementary Material Figure 1: Structured CPAP Weaning Protocol – Flowchart

Universität Hamburg
Universitätsklinikum Hamburg-Eppendorf

No CPAP pauses if

- GA ≤ 29+0 weeks
- PEEP > 5.0 cmH_2O
- Within 24 h after LISA
- GA ≤ 34+6 weeks and within 24 h after extubation

Stage 1: 15–20 min CPAP-off (nursing round)

Stage 2: ~1 h CPAP-off (kangaroo care/prone)

Stage 3: 1–3 h CPAP-off per shift

Stage 4: Up to 6 h CPAP-off

Stage 5: CPAP-off daytime, CPAP at night if required

Stage 6: Complete discontinuation of CPAP

GA 29+0 to 30+6 weeks
Start with **Stage 1**

GA >= 31 + 0 weeks
Start with **Stage 3**

"Stable?" at each pause and every 24 hours
Stable / tolerable → proceed to next stage
Unstable → remain at current stage

Parameter	Stable	Tolerable	Unstable
Oxygen requirement	21% / 0 $L \cdot min^{-1}$	22–25% or 0.1–0.5 $L \cdot min^{-1}$	>25% or >0.5 $L \cdot min^{-1}$
Respiratory rate	30–60 min^{-1}	<60 or 60–100 (feeding excluded)	>100 (feeding excluded)
Work of breathing	Normal	Mild retractions, nasal flaring, periodic breathing	≥2 signs, grunting, seesaw breathing
Events during CPAP pause (per h)	<3 severe or <10 mild	3 severe or 10 mild	>3 severe or >10 mild
