# Supplementary material for: Structured continuous positive airway pressure weaning standardizes discontinuation and reduces instability events
Source: Front Pediatr. 2026 Mar 11;14:1776103. doi: 10.3389/fped.2026.1776103 (PMC13013428; doi:10.3389/fped.2026.1776103)

Supplementary Material Figure 2: Monitor Card

Universitätsklinikum Hamburg-Eppendorf

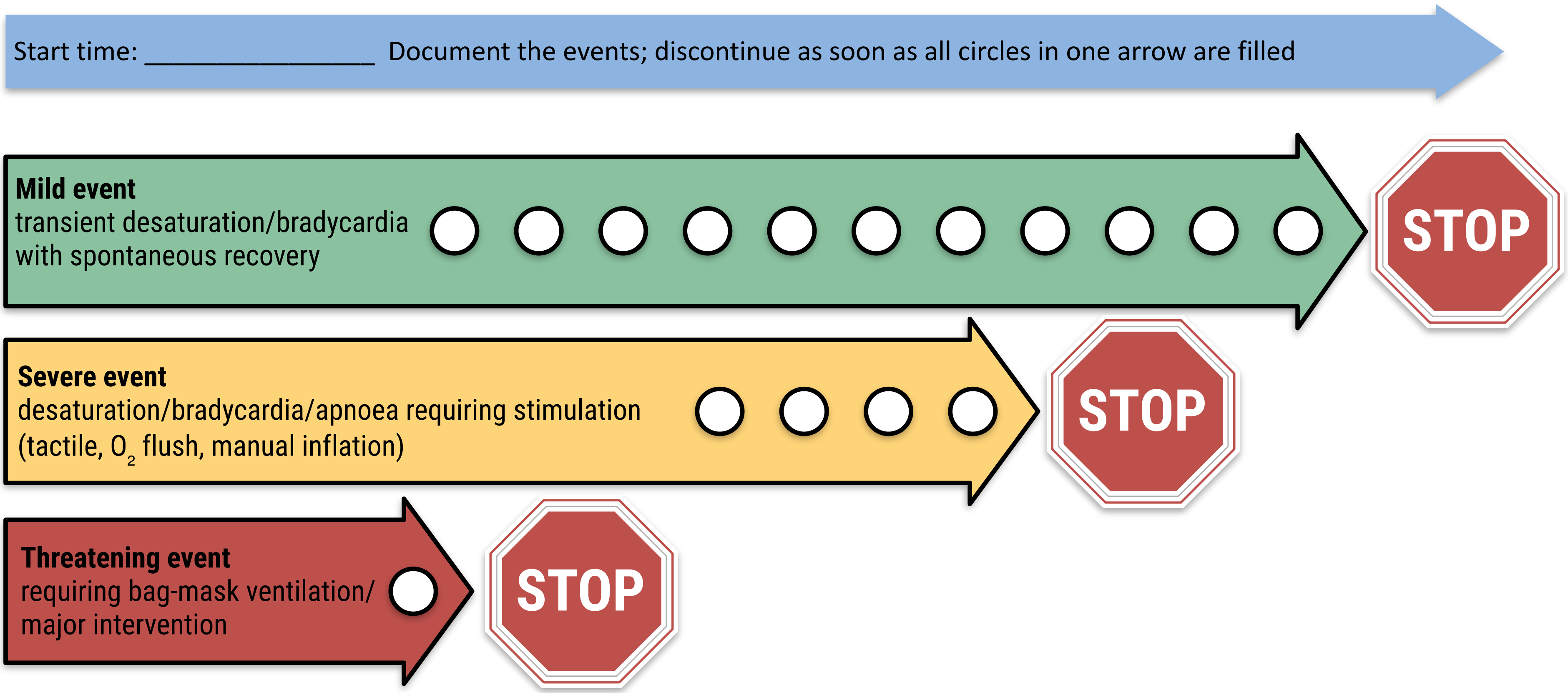

Supplement: Supplementary file 3 [file Image2.pdf]
